# The Impact of Frailty and Gender Differences on Hospitalization and Complications in Proximal Femoral Pathological Fractures: A Cross-Sectional Study

**DOI:** 10.3390/jpm14090991

**Published:** 2024-09-18

**Authors:** Alessandro El Motassime, Elisa Pesare, Andrea Russo, Sara Salini, Giordana Gava, Carla Recupero, Tommaso Giani, Marcello Covino, Giulio Maccauro, Raffaele Vitiello

**Affiliations:** 1 Department of Ageing, Neurosciences, Head-Neck and Orthopaedics Sciences, Università Cattolica del Sacro Cuore, 00168 Rome, Italy or alessandro.elmotassime01@icatt.it (A.E.M.); giordana.gava01@icatt.it (G.G.); carla.recupero01@icatt.it (C.R.); tommaso.giani01@icatt.it (T.G.); giulio.maccauro@policlinicogemelli.it (G.M.); raffaele.vitiello@guest.policlinicogemelli.it (R.V.); 2 Orthopedic Unit, Department of Traslational Biomedicine and Neuroscience DiBraiN, Policlinico di Bari, 70124 Bari, Italy; 3 Fondazione Policlinico Gemelli, 00168 Rome, Italy; andrea.russo1@policlinicogemelli.it (A.R.); sara.salini@pliclinicogemelli.it (S.S.); 4 Department of Emergency, Anesthesiological and Reanimation Sciences, Università Cattolica del Sacro Cuore, 00168 Rome, Italy; marcello.covino@policlinicogemelli.it

**Keywords:** gender, frailty, pathological fractures, femur fractures

## Abstract

Background: Frailty associated with aging increases the risk of falls, disability, and death. The aim of this study is to explore gender-related disparities in the survival outcomes of pathological femoral fractures in older frail patients, while analyzing potential specific prognostic factors. Methods: This study is a retrospective observational analysis conducted at a single medical center. It enrolled all patients aged 65 and above who were admitted to our emergency department between 2016 and 2020 with a diagnosis of pathological femur fracture requiring surgical intervention. The primary study endpoint was evaluating gender-related differences in survival outcomes. The secondary endpoint involves investigating gender-specific prognostic factors through the analysis of clinical and laboratory parameters. Results: The average Charlson Comorbidity Index (CCI) was slightly lower in men, but the difference was not statistically significant (*p* = 0.53). The Clinical Frailty Scale (CFS) showed similar results, with men and women 5.23 (SD 1.46), also not significant (*p* = 0.83). An evaluation comparing patients aged 75 years or younger to those older than 75 years found significant differences in health metrics. The average CCI was higher in the over 75 group compared to the under 75 group, with a *p*-value of 0.001. Similarly, the CFS average was also greater in the over 75 group than in the under 75 group, with a *p*-value of 0.0001. Complications were more frequent in patients over 75 and those with lower educational qualifications. The evaluation analyzed cardiac patients compared to a control group, revealing that the average age of cardiac patients was 75.22 years, while the control group was younger at 73.98 years (*p* = 0.5119). The CCI for cardiac patients averaged 6.53, significantly higher than 4.43 for non-cardiac patients (*p* = 0.0003). Conclusion: Frailty assessment is therefore essential in patients with pathological fracture of the proximal femur and is an important predictor of both gender differences and hospital complications. Enhancing gender analysis in this field is crucial to gather more robust evidence and deeper comprehension of potential sex- and gender-based disparities.

## 1. Introduction

Proximal femur fractures are widely recognized as a significant health and social issue [1,2] due to their substantial social costs and their detrimental impact on patient mortality [3,4,5]. With the average life expectancy increasing significantly in recent years, a rapid aging of the population is underway [6]. It is projected that approximately 17% of the global population will be over 65 years old by 2050 [7], contributing to an estimated doubling of proximal femoral fracture incidence by that time.

Consequently, there is a growing emphasis on the proper management of patients with proximal femur pathological fractures [8].

Indeed, owing to its robust mechanical structure and well-developed vascular network, the proximal femur emerges as a frequent site for skeletal metastasis, with approximately 60% of impending or actual pathologic fractures occurring in this region [9]. Among malignancies originating from the breast, prostate, lung, kidney, and colon, bone metastases are particularly prevalent, with the majority (excluding prostate cancer) exhibiting lytic or mixed characteristics [9,10]. Notably, close to 10% of these malignant tumors will metastasize to the proximal femur [11,12]. Regarding localization within the femur, roughly 50% of lesions manifest in the femoral neck, followed by 30% in the subtrochanteric region and 20% in the intertrochanteric region [9].

Moreover, the extended life expectancy of oncological patients, coupled with the imperative to preserve functionality and quality of life [12], has led to a heightened influx of such patients into emergency departments [11,12]. Many of these individuals are frail and burdened with multiple comorbidities.

In these instances, treatment must be customized on an individual basis, taking into account various clinical factors, estimated life expectancy, and the overall condition of each patient [13]. The literature indicates a correlation between mortality and frailty that remains independent of other clinical considerations [1,14]. Frailty can generally be characterized as a multidimensional state of heightened vulnerability to both internal and external stressors [15]. Often associated with aging, frailty exacerbates the risks of falls, disability, and mortality [16].

Presently, there is a growing emphasis on analyzing multiple risk factors, complication [17] rates, and surgical interventions in this field [18]. Stratifying patients based on risk class is crucial for evaluating prognosis accurately.

Moreover, gender considerations are now being given greater attention [19,20]. Historically, they have often been limited to demographic data (with females being more affected than males) [19]. However, comprehending how pathological femoral fractures affect male and female patients differently is vital for optimizing treatments and rehabilitation [21] protocols during hospitalization [22], as well as for effective communication with patients and their families [23]. Closing this research gap would facilitate the development of gender-sensitive strategies in prevention, diagnosis, and treatment [19].

The aim of this study is to explore gender-related disparities in the survival outcomes of pathological femoral fractures in older frail patients, while analyzing potential specific prognostic factors.

## 2. Materials and Methods

### 2.1. Study Design and Inclusion Criteria

This study is a retrospective observational analysis conducted at a single medical center. It enrolled all patients aged 65 and above who were admitted to our emergency department between 2016 and 2020 with a diagnosis of pathological femur fracture requiring surgical intervention. Prior to surgery, patients underwent oncological staging to assess the primary lesion and determine the presence of visceral or multiple bone metastases. All patients had their primary lesions identified before undergoing surgery. Inclusion criteria comprised pathological fractures of the femur confirmed by histological examination. Exclusion criteria included primary bone tumors, impending fractures, open fractures, fractures in patients under 65 years of age, and those who were transferred to other institutions or who declined hospitalization. Informed consent was waived due to the retrospective and anonymized nature of the study design.

### 2.2. Study Variables

All patients attended an initial preoperative assessment appointment and underwent standard blood tests before surgery. Upon recovery, data on gender, age, type of fracture, time waited before surgery, duration of hospital stay, and comorbidities such as dementia, diabetes mellitus, obesity, mild to severe liver disease, chronic pulmonary disease, and cardiovascular conditions (including ischemic heart disease, stroke, congestive heart failure, and peripheral artery disease) were collected. The Charlson Comorbidity Index was calculated based on preexisting comorbidity data.

Clinical postoperative complications, such as infections at the surgical site, pneumonia, urinary tract infections, heart attacks, fever, sepsis, and postoperative anemia, were documented.

Blood tests performed before surgery were repeated on the first and third days following surgery, and subsequently as required according to patients’ needs. Laboratory values and their trends were recorded. We gathered laboratory values and their trends, including hemoglobin levels, platelet counts, white blood cell counts, fibrinogen levels, and creatinine levels.

### 2.3. Study Endpoints

The primary study endpoint was evaluating gender-related differences in survival outcomes, as well as identifying risk factors for hospital mortality. The secondary endpoint involves investigating gender-specific prognostic factors through the analysis of clinical and laboratory parameters.

### 2.4. Ethical Approval 

The study was conducted according to the principles expressed in the Declaration of Helsinki and its later amendments. The research protocol was approved by the Institutional Review Board of Fondazione Policlinico Universitario “A Gemelli” IRCCS–Rome (#0025817/22; Study ID: #5121.)

## 3. Results

In our study on pathological fractures at the emergency department of Policlinico Gemelli, Rome, we examined gender differences, lifestyle habits, and clinical presentation to identify patterns for personalized diagnosis and treatment. From 1 January 2016 to 1 January 2020, we admitted 103 patients with suspected pathological fractures, all of whom underwent surgical treatment. Biopsies confirmed pathological fractures in 61 patients. We analyzed various characteristics to classify the patients into different groups.

In terms of gender, 22 patients were men and 39 were women; none were transgender. The average age was 74.3 years (SD 6.29), with men averaging 74.86 years (SD 7.04) and women averaging 73.97 years (SD 5.89).

Upon arrival at the emergency department, all patients were interviewed about their habits, medical history, and existing conditions. The analysis of the patients’ medical history revealed the following: 15 patients had cardiological conditions, 11 of whom were on anticoagulant therapy, 5 had hematological disorders, 3 had diabetes, and another 3 had pulmonary diseases.

At the end of the initial interview to evaluate the overall condition, the Charlson Comorbidity Index (CCI) was calculated, resulting in an average of 5.35 (SD 2.05), and the Clinical Frailty Scale (CFS) was calculated, resulting in an average of 5.2 (SD 1.58).

After the data were collected, it was found that 25 patients (14 men, 11 women) had a history of smoking, while 4 patients lived in residential facilities, and the remaining 57 lived in their own homes.

We also examined the educational background of the patients under evaluation. We found that 10 had completed elementary school, 25 had finished middle school, 15 had obtained a high school diploma, and 11 had a university degree. Shortly after admission, the patients underwent blood tests, yielding the following average results: hemoglobin 11.33 g/dL (SD 2.08), blood glucose 126.89 mg/dL (SD 31.63), creatinine 1 mg/dL (SD 1.03), fibrinogen 466.84 mg/dL (SD 154.73), white blood cells 7.5 × 10^9^/dL (SD 2.12), and platelets 288.82 × 10^3^/mm^3^ (SD 118.38).

Upon analysis of the results, it was found that none of the patients admitted to the emergency department had liver or kidney diseases, a history of stroke, peripheral vascular diseases or dementia, connective tissue diseases, or HIV at the time of admission.

The average time required in the emergency department to stabilize the patient clinically before performing the surgical procedure and biopsy was also evaluated, resulting in an average of 63.17 h (SD 53.95). Subsequently, the biopsy samples were sent to the pathology center for histological examination. Once the histological results were obtained, the findings were as follows: 18 breast carcinomas, 11 lung carcinomas, 9 kidney carcinomas, 5 prostate carcinomas, 3 multiple myelomas, 3 pancreatic carcinomas, 3 uterine carcinomas, 3 thyroid carcinomas, 1 chondrosarcoma, 1 colon carcinoma, 1 gastric carcinoma, 1 vulvar carcinoma, 1 bladder carcinoma, and 1 Hodgkin lymphoma.

After evaluating the general group, further assessments were conducted on possible subgroups by dividing the patients based on the following parameters: gender, age, smoking habit in the last 5 years, education, positive history of heart disease, and primary tumor. 

From the evaluation of the different results between the group of men, consisting of 22 patients, and the group of women, consisting of 39 patients, it was found that the average CCI was 4.73 (SD 2.07) in men and 5.08 (SD 2.06) in women (*p* = 0.53), while the CFS was 5.14 (SD 1.81) in men and 5.23 (SD 1.46) in women (*p* = 0.83). Regarding the average length of stay in the emergency department, the values were 31.19 h (SD 28.5) for men and 28.14 h (SD 17.18) for women (*p* = 0.60), while the overall length of stay from admission to the emergency department was 17.66 days (SD 10.85) for men and 18.82 days (SD 19.94) for women (*p* = 0.80).

Both groups had only 1 death during hospitalization, which corresponds to an average of 0.05 in men and 0.026 in women. Regarding complications, we observed 4 complications in men and 10 in women, corresponding to an average of 0.18 in men and 0.26 in women. Table 1

Another evaluation was conducted to compare the group of patients aged 75 years or younger with the group of patients older than 75 years. This evaluation revealed that the average CCI was 5.8 (SD 1.92) in the over 75 group and 4.13 (SD 1.86) in the under 75 group (*p* = 0.001), while the CFS was 6.13 (SD 1.36) in the over 75 group and 4.29 (SD 1.22) in the under 75 group (*p* = 0.0001). Regarding the average length of stay in the emergency department, the values were 30 h (SD 24,82) in the over 75 group and 28.5 h (SD 18.74) in the under 75 group (*p* = 0.79), while the overall length of stay from admission to the emergency department was 18.82 days (SD 19.08) in the over 75 group and 17.99 days (SD 15.31) in the under 75 group (*p* = 0.85).

The incidence of complications in the over 75 group was 11, while in patients younger than 75 years, there were only 3 complications (*p* = 0.016). Table 2

The patients were then divided and evaluated based on their level of education. The resulting groups were: 10 patients with an elementary school diploma, 25 patients with a middle school diploma, 15 patients with a high school diploma, and 11 patients with a university degree or higher qualifications. We then decided to compare the group of 26 patients with a high school diploma or higher with the remaining 35 patients. The average CCI values were 4 (SD 1) for the group with a high school diploma or higher, 4.43 (SD 2.07) for the patients with a lower degree (*p* = 0.33), while the average CFS values were 5.8 (SD 0.84) for the group with a high school diploma or higher, 5.14 (SD 2.03) for the patients with a lower degree (*p* = 0.12).

The average length of stay in the emergency department was 45.5 (SD 41.72) for the group with an elementary school diploma, 34.61 (SD 42.37) for the patients with a middle school diploma, 17.36 (SD 4.95) for the patients with a high school diploma, and 6 (SD 1.2) for the patients with a university degree.

The incidence of complications in the various groups was six for the group with an elementary school diploma, four for the patients with a middle school diploma, two for the patients with a high school diploma, and two for the patients with a university degree.

This resulted in a total of 4 complications in the group with a high school diploma or higher, while there were 10 complications in the remaining group (*p* = 0.35).

Similarly, the patients considered in the study were divided based on the presence or absence of cardiac pathology, resulting in a group of 15 cardiac patients compared to the remaining 46.

From this evaluation, it emerged that in the group of cardiac patients, the average age was 75.22 years (SD 6.32) compared to 73.98 years (SD 6.32) (*p* = 0.5119). From the assessment of comorbidities, it was found that the average CCI of the cardiac patients was 6.53 (SD 1.68) compared to 4.43 (SD 1.9) for the others (*p* = 0.0003), while the CFS of the cardiac patients was 5.8 (SD 1.37) compared to 5 (SD 1.6) (*p* = 0.0875).

The evaluation of the length of stay in the emergency department showed that the cardiac patients had an average stay of 32.09 h (SD 23.56) compared to 28.31 h (SD 21.33) for the non-cardiac patients (*p* = 0.5458). Subsequently, the average hospitalization times were compared, resulting in 15.27 days (SD 7.58) for the cardiac patients versus 19.42 days (SD 19.2) for the non-cardiac patients (*p* = 0.4195).

Overall, there were 14 complications, with 5 in the cardiac patient group and 9 in the others, while smoking habits were found in three cardiac patients and in nine of the others. Table 3

The patients were then categorized based on their geographic area of origin and divided between those coming from metropolitan areas and those from rural areas. As a result of this division, we found that the group of patients from the city consisted of 40 individuals with an average age of 75 years (SD 6.57), while the remaining 21 patients were from rural areas with an average age of 72.95 years (SD 5.61) (*p* = 0.07). The comorbidities of the patients were also evaluated, resulting in an average CCI of 4.7 (SD 1.76) for city residents and 5.43 (SD 2.5) for rural residents (*p* = 0.18). The CFS was 5.1 (SD 1.53) for city patients and 5.38 (SD 1.69) for rural patients (*p* = 0.51). The average time spent in the emergency room was 30.1 h (SD 21.69) for city patients and 27.61 h (SD 22.34) for rural patients (*p* = 0.6748). The average hospitalization duration was 19.5 days (SD 17.82) for city patients and 16.31 days (SD 15.93) for rural patients (*p* = 0.49). Lastly, we evaluated the complications, which were eight in the city patient group and six in the rural patient group (*p* = 0.52). Table 4

## 4. Discussion

Proximal femur fractures are a significant concern due to their substantial economic and social impact [4]. These fractures have profound consequences for patients’ quality of life, requiring a multidisciplinary approach to provide the best possible care and achieve faster rehabilitation [22].

This scenario gains even greater importance when dealing with oncology patients with pathological fractures [23].

To the best of the authors’ knowledge, the literature contains numerous studies exploring factors associated with PFF and their care [1,2,24]; however, there is a lack of studies investigating whether sex or other characteristics may be one of these factors.

Sex- and gender-related factors play a significant role in health across the lifespan, affecting disease development, responses to medications and surgeries [19], and overall clinical outcomes [25].

Sex refers to the biological differences between females and males, including reproductive anatomy, hormone levels, gene expression, and physiological and anatomical characteristics [7].

Gender is a complex concept influenced by non-biological factors like education, culture, psychology, economics, medication use, lifestyle, health conditions, and religious beliefs [26,27,28].

The current orthopedic literature has emphasized the lack of research addressing gender- and sex-related factors in the study of pathological PFF.

Our study aimed to perform an epidemiological analysis of the characteristics of patients who access the emergency department for a pathological fracture of the proximal femur. A more in-depth analysis of the data revealed that for seven patients, the initial diagnosis of neoplasia was made due to the biopsy performed for the suspected pathological fracture. This highlights the importance of early diagnosis. Emphasis should be placed on screening, especially for elderly patients, as the youngest patient was 65 years old. In this age group, regular check-ups can be crucial for the early detection of chronic and particularly neoplastic diseases.

Similarly, great importance should be given to the collection of a thorough medical history, allowing an accurate assessment of the potential causes of the fracture, especially in patients who do not report recent traumatic episodes upon accessing the emergency department. Regarding the management of these patients, the creation of dedicated diagnostic and therapeutic pathways is crucial. A multidisciplinary approach involving oncologists, internists, and surgeons is essential to provide the highest quality of care [10].

A thorough analysis of the patient characteristics allowed us to identify several variables with a *p* value < 0.05. The variables that are statistically significant are: in the comparison between cardiac and non-cardiac patients, CCI stands out, while in the comparison between patients under and over 75, both CCI and CFS are noteworthy; furthermore, in the comparison between patients under and over 75, the evaluation of complications occurring during hospitalization resulted in a *p* value of 0.0159, making the result statistically significant. None of the other variables considered in the different groups met the selected criteria. However, these variables require further evaluation to confirm the statistical significance of CCI and CFS in the specified categories and to verify the lack of statistical relevance of the other variables under consideration.

We believe that this study can set the stage for a more thorough analysis of the factors that define and can impact clinical progression, with the goal of achieving increasingly personalized medicine. However, we acknowledge the primary limitations of this article; namely, the small sample size and the fact that it was conducted at a single center.

## 5. Conclusions

To the authors’ best knowledge, the orthopedic literature lacks studies on gender, particularly within the context of oncological patients. In this scenario, the authors believe that despite the mentioned limitations, this article can serve as a starting point. The topic deserves further investigation with a greater focus on detail to provide personalized care.

Enhancing gender analysis in this field is crucial to gather more robust evidence and deeper comprehension of potential sex- and gender-based disparities.

## Figures and Tables

**Table 1:** Male vs. female categorization.

Sex	N°	Age	CCI	CFS	LOS	Complications
Male	22	74.86	4.73	5.14	17.66	4
Female	39	73.97	5.08	5.23	18.82	10

**Table 2:** Under 75 vs. over 75 categorization.

Age	N°	CCI	CFS	LOS	Complications
Under 75	31	4.13	4.29	17.99	3
Over 75	30	5.8	6.13	18.82	11

**Table 3:** Categorization based on the presence or absence of cardiac pathology.

Cardiac Pathology	N°	Age	CCI	CFS	LOS	Complications
Yes	15	75.22	6.53	5.8	15.27	5
No	46	73.98	4.43	5	19.42	9

**Table 4:** Categorization based on geographic area of origin.

Geographic Area	N°	Age	CCI	CFS	LOS	Complications
City	40	75	4.7	5.1	19.5	8
Rural	21	72.95	5.43	5.38	16.31	6

## Data Availability

The datasets used and/or analyzed during the current study are available from the corresponding author upon reasonable request.
